# Evolutionary dynamics in structured populations

**DOI:** 10.1098/rstb.2009.0215

**Published:** 2010-01-12

**Authors:** Martin A. Nowak, Corina E. Tarnita, Tibor Antal

**Affiliations:** Program for Evolutionary Dynamics, Department of Mathematics, Department of Organismic and Evolutionary Biology, Harvard University, Cambridge, MA 02138, USA

**Keywords:** evolutionary game theory, spatial games, evolutionary graph theory, games on sets, evolution of cooperation, spatial selection

## Abstract

Evolutionary dynamics shape the living world around us. At the centre of every evolutionary process is a population of reproducing individuals. The structure of that population affects evolutionary dynamics. The individuals can be molecules, cells, viruses, multicellular organisms or humans. Whenever the fitness of individuals depends on the relative abundance of phenotypes in the population, we are in the realm of evolutionary game theory. Evolutionary game theory is a general approach that can describe the competition of species in an ecosystem, the interaction between hosts and parasites, between viruses and cells, and also the spread of ideas and behaviours in the human population. In this perspective, we review the recent advances in evolutionary game dynamics with a particular emphasis on stochastic approaches in finite sized and structured populations. We give simple, fundamental laws that determine how natural selection chooses between competing strategies. We study the well-mixed population, evolutionary graph theory, games in phenotype space and evolutionary set theory. We apply these results to the evolution of cooperation. The mechanism that leads to the evolution of cooperation in these settings could be called ‘spatial selection’: cooperators prevail against defectors by clustering in physical or other spaces.

## 1. Introduction

An evolving population consists of reproducing individuals, which are information carriers. When they reproduce, they pass on information. New mutants arise if this process involves mistakes. Natural selection emerges if mutants reproduce at different rates and compete for limiting resources. The two most important media for carrying forward the information of the evolutionary processes on Earth are biological polymers (such as DNA and RNA) and human language. The first gives rise to genetic evolution and the second to cultural evolution. The mathematical approaches that we discuss below can be interpreted within either of these two domains. There is also a non-linguistic cultural evolution: we can imitate behavioural patterns without talking about them.

Evolution has become a discipline with a rich mathematical formalism. The mathematical description of evolutionary processes is helpful for a rigorous understanding. We do not see the mathematical approach to evolutionary dynamics as a metaphor, but as a description of biological reality. Life unfolds according to the mathematical laws of evolution.

Constant selection means that the fitness values of individuals are constant and do not depend on the composition of the population. Frequency-dependent selection means that the fitness values depend on the relative abundances (=frequencies) of various types in the population. Constant selection can be seen as a population adapting on a fixed fitness landscape (Eigen & Schuster 1977, 1978), while frequency-dependent selection implies that the population changes the fitness landscape as it moves over it (Nowak & Sigmund 2004).

Frequency-dependent selection brings us into the world of evolutionary game theory (Maynard Smith 1982; Hofbauer & Sigmund 1988). Evolutionary game theory was originally designed as a tool for studying animal behaviour (Maynard Smith & Price 1973; Houston & McNamara 1999) but has become a general approach that transcends almost every aspect of evolutionary biology (Nowak & Sigmund 2004). Evolutionary game dynamics include the competition of species in an ecosystem (May 1973; May & Leonard 1975), the evolution of virulence in host–parasite interactions (Levin & Pimentel 1981; May & Anderson 1983; Bonhoeffer & Nowak 1994; Nowak & May 1994), the interaction between viruses and cells of the immune system (Nowak *et al.* 1995; Nowak & May 2000), the competition between phages for bacterial cells (Turner & Chao 1999), the evolution of metabolic pathways (Pfeiffer *et al.* 2001) and the evolution of human language (Nowak *et al.* 2002).

The dynamical interactions in any group of humans with bonding, economic exchanges, learning from each other and exploration of new strategies represent evolutionary games. Classical game theory was invented as a mathematical tool for studying economic and strategic decisions of humans (Von Neuman & Morgenstern 1944; Luce & Raiffa 1957; Fudenberg & Tirole 1991; Osborne & Rubinstein 1994; Skyrms 1996; Samuelson 1997; Binmore 2007). Evolutionary game theory has added the concept of a population of players and the idea that the payoff is interpreted as fitness. These two concepts naturally lead to a dynamical approach (Maynard Smith 1982; Hofbauer & Sigmund 1988, 1998, 2003; Weibull 1995; McNamara *et al.* 1999; Michod 1999; Gintis 2000; Hauert *et al.* 2002, 2007; Cressman 2003; Nowak 2006*a*).

The traditional framework of evolutionary game theory rests on differential equations, which describe deterministic dynamics in well-mixed and infinitely large populations. At the centre of this endeavour is the so-called ‘replicator equation’ (Taylor & Jonker 1978; Hofbauer *et al.* 1979; Zeeman 1980), where *x*_*i*_ is the frequency and *f*_*i*_ = *∑*_*i*_ *a*_*ij*_ *x*_*j*_ is the fitness of strategy *i*. The coefficients, *a*_*ij*_, are the elements of the payoff matrix. The replicator equation is given by d*x*_*i*_/d*t* = *x*_*i*_(*f*_*i*_ − *f̄*), where *f̄* is the average fitness of the population. The replicator equation is linked to the concept of a Nash equilibrium (Nash 1950). If strategy *i* is a strict Nash equilibrium, which means that *a*_*ii*_ > *a*_*ji*_ for all *j* ≠ *i*, then it is an asymptotically stable fixed point of the replicator equation (Hofbauer & Sigmund 1988). A strict Nash equilibrium is similar to an evolutionarily stable strategy (ESS).

Infinitely large, well-mixed populations and deterministic dynamics are idealizations. Real populations have a finite number of individuals and are not well mixed. Typically, it is not the case that any two individuals interact with the same probability. For example, the spatial distribution of a population makes interactions among neighbours more likely than interactions between distant individuals. The social network in human populations causes friends to interact more often than strangers. These realizations led to spatial approaches for evolutionary game dynamics (Nowak & May 1992, 1993; Ellison 1993; Herz 1994; Lindgren & Nordahl 1994; Ferriere & Michod 1996; Killingback & Doebeli 1996; Nakamaru *et al.* 1997, 1998; Szabó & Tőke 1998; van Baalen & Rand 1998; Hofbauer 1999; Szabó *et al.* 2000; Hutson & Vickers 2002; Kerr *et al.* 2002; Hauert & Doebeli 2004; Yamamura *et al.* 2004; Nakamaru & Iwasa 2005; Helbing & Yu 2008) and later to evolutionary graph theory (Lieberman *et al.* 2005; Ohtsuki & Nowak 2006*a*,*b*; Ohtsuki *et al.* 2006). Spatial models have a long tradition in ecology (Levin & Paine 1974; Durrett & Levin 1994*a*,*b*; Hassell *et al.* 1994; Tilman & Kareiva 1997), and they have also been analysed with the methods of inclusive fitness theory (Hamilton 1964; Seger 1981; Grafen 1985, 2006; Taylor 1992; Rousset & Billiard 2000; Taylor *et al.* 2000; Rousset 2004).

Evolutionary dynamics in finite-sized populations are not deterministic but stochastic. If two mutants have exactly the same fitness, eventually one of them will take over, while the other becomes extinct. An advantageous mutant has a certain probability to win, but no certainty. Sometimes deleterious mutants can prevail, thereby allowing the evolutionary process to cross fitness valleys.

These considerations bring us to some of the great open questions in the field. How can we formulate stochastic evolutionary (game) dynamics in populations of finite size? How does natural selection choose between strategies in structured populations? What does evolutionary stability mean in structured populations and in the presence of random drift? What is a general description of population structure? For some of these questions, we suggest answers in this article.

We apply the results presented in this paper to the evolution of cooperation, which is a fascinating topic in evolutionary biology (Trivers 1971; Axelrod & Hamilton 1981; May 1987; Milinski 1987; Nowak & Sigmund 1990, 2005; Doebeli & Knowlton 1998; Frank 1998; Bshary *et al.* 2008). How does natural selection lead to situations where competing individuals help each other? Cooperation is important because it allows construction. Without cooperation, there is no tendency in evolution to lead to ever increasing complexity. New levels of organization emerge, because competing entities learn to cooperate. For that reason, one can argue that cooperation is a third fundamental principle of evolution, next to mutation and selection (Nowak 2006*b*).

This article is arranged as follows. In §2, we discuss strategy selection in well-mixed populations. In §3, we present the concept of ‘structural dominance’ and introduce the ‘structure coefficient’, *σ*. In §4, we discuss evolutionary graph theory. In §5, we study ‘games in phenotype space’. In §6, we discuss evolutionary set theory. In §7, we apply the results of the previous sections to the evolution of cooperation. Section 8 offers a conclusion.

## 2. Evolutionary games in well-mixed populations

In a well-mixed population, any two individuals interact with the same probability. The well-mixed population is the reference point for any analysis of how population structure affects evolution. Therefore, we begin by studying strategy selection in the well-mixed population. For all subsequent models, the well-mixed population always represents a special case. For example, in evolutionary graph theory the well-mixed population is given by a complete graph with identical weights. In evolutionary set theory, the well-mixed population is obtained if all individuals are in the same set.

### (a) Two strategies

Consider a game between two strategies, *A* and *B*. If two *A* players interact, both get payoff *a*; if *A* interacts with *B*, then *A* gets *b* and *B* gets *c*; if two *B* players interact, both get *d*. These interactions are represented by the payoff matrix2.1
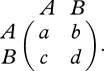

We consider a population of size *N*, in which there are *i* individuals of type *A* and *N* − *i* of type *B*. The variable, *i*, ranges from 0 to *N*. For an *A* individual, there are *i* − 1 other *A* individuals. For a *B* individual, there are *N* − *i* − 1 other *B* individuals. Therefore, the expected payoffs are *F*_*A*_ = [*a*(*i* − 1) + *b*(*N* − *i*)]/(*N* − 1) and *F*_*B*_ = [*ci* + *d*(*N* − *i* − 1)]/(*N* − 1).

Payoff translates into reproductive success. Here, we assume that fitness is a linear function of payoff: *f*_*A*_ = 1 + *wF*_*A*_ and *f*_*B*_ = 1 + *wF*_*B*_. The constant, 1, represents the ‘background fitness’ which is independent of the game. The parameter *w* denotes the intensity of selection; it quantifies how strongly the particular game under consideration affects the fitness of individuals. The limit *w* → 0 represents weak selection. Many analytical insights can be derived for this limit, because weak selection tends to linearize the involved functions (Nowak *et al.* 2004; Taylor *et al.* 2004; Tarnita *et al.* 2009*a*).

For each updating step, we pick one individual for death at random and one individual for birth proportional to fitness. The offspring of the second individual replaces the first. Hence, the total population size is strictly constant. This stochastic process was introduced by Moran (1958) for the study of constant selection. We can also interpret the individual update steps as learning events. At random, an individual decides to update its strategy. He picks a ‘teacher’ from the population proportional to fitness and tries to imitate her strategy. Let us now add mutation. With probability 1 − *u*, the strategy of the parent (or teacher) is adopted, but with probability *u*, one of the two strategies (*A* or *B*) is chosen at random. The mutation rate *u* is a parameter between 0 and 1.

We find that *A* is more abundant than *B* in the stationary distribution of the mutation–selection process if2.2
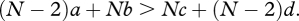



This condition was first derived by Kandori *et al.* (1993) for low mutation in an evolutionary process that is deterministic in following the gradient of selection. Nowak *et al.* (2004) obtained this result for a stochastic selection process by comparing the two fixation probabilities, *ρ*_*A*_ and *ρ*_*B*_, in the limit of weak selection. Antal *et al.* (2009*a*) showed that condition (2.2) holds for a large variety of stochastic mutation–selection processes for any intensity of selection and any mutation rate.

For large population size, *N*, we obtain *a* + *b* > *c* + *d*, which is the well-known condition for risk dominance in a coordination game (Harsanyi & Selten 1988). A coordination game is defined by *a* > *c* and *b* < *d*. In this case, both *A* and *B* are Nash equilibria. The risk dominant equilibrium has the bigger basin of attraction. The Pareto efficient equilibrium has the higher payoff. For example, if *a* + *b* > *c* + *d* then *A* is risk-dominant, but if *a* < *d* then *B* is Pareto efficient. It is often interesting to ask when Pareto efficiency is chosen over risk dominance.

### (b) Two or more strategies

Let us now consider a game with *n* strategies. The payoff values are given by the *n × n* payoff matrix *A* = [*a*_*ij*_]. This means that an individual using strategy *i* receives payoff *a*_*ij*_ when interacting with an individual that uses strategy *j*.

We consider the same evolutionary process as before. Mutation means, with probability *u*, one of the *n* strategies is chosen at random. Let us introduce the parameter *μ* = *Nu*, which is the rate at which the entire population produces mutants. We say that selection favours strategy *k* if the average abundance of *k* is greater than 1/*n* in the stationary distribution of the mutation–selection process. The following results were derived by Antal *et al.* (2009*b*) and hold for weak selection and large population size.

For low mutation, *μ* → 0, the population almost always consists of only a single strategy. This strategy is challenged by one invading strategy at a time. The invader becomes extinct or takes over the population. Thus, the crucial quantities are the ‘pairwise dominance measures’, *a*_*ii*_ + *a*_*ij*_ − *a*_*ji*_ − *a*_*jj*_. It turns out that selection favours strategy *k* if the average over all pairwise dominance measures is positive,2.3
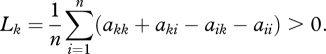



For high mutation, *μ* → ∞, the population contains each strategy at roughly the same frequency, 1/*n*, at any time. The average payoff of strategy k is *ā*_*k*_ = ∑_*j*_ *a*_*kj*_/*n*, while the average payoff of all strategies is *ā* = ∑_*j*_ *ā*_*j*_/*n*. Strategy *k* is favoured by selection if its average payoff exceeds that of the population, *ā*_*k*_ > *ā*. This condition can be written as2.4
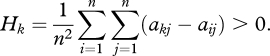



We note that this condition holds for large mutation rate and any intensity of selection.

Amazingly, for any mutation rate, strategy *k* is favoured by selection if a simple linear combination of equations (2.3) and (2.4) holds,2.5
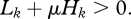



Moreover, in the stationary distribution, *k* is more abundant than *j* if2.6
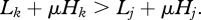



Equations (2.5) and (2.6) are useful conditions that quantify strategy selection for *n* × *n* games in well-mixed populations. They hold for weak selection, large population size, but any mutation rate. Equations (2.3)–(2.6) can also be generalized to continuous strategy spaces and mixed strategies (Tarnita *et al.* 2009*c*).

## 3. Structural dominance

Before we turn to specific approaches for exploring population structure, we present a general result that holds for almost any processes of evolutionary game dynamics in well-mixed or structured populations. Consider two strategies, *A* and *B*, and the payoff matrix (2.1). Tarnita *et al.* (2009*a*) showed that for weak selection, the condition that *A* is more abundant than *B* in the stationary distribution of the mutation–selection process can be written as a linear inequality in the payoff values3.1
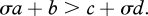



The parameter, *σ*, which we call ‘structure coefficient’, can depend on the population structure, the update rule, the population size and the mutation rate, but does not depend on *a*, *b*, *c*, *d*. Therefore, the effect of population structure can be summarized by a single parameter, *σ*, if we are only interested in the question which of the two strategies, *A* or *B*, is more abundant in the stationary distribution of the mutation–selection process in the limit of weak selection.

For a large well-mixed population, we have *σ* = 1; see §2*a*. But in structured populations, we can obtain *σ* > 1. In this case, the diagonal entries of the payoff matrix are more important than the off-diagonal entries for determining strategy selection. This property allows selection of Pareto efficiency over risk dominance in coordination games. It also allows the evolution of cooperation, as we will see in §7.

In the subsequent sections, the crucial results will be expressed as *σ* values. These *σ* values quantify how natural selection chooses between competing strategies for particular population structures and update rules.

## 4. Spatial games and evolutionary graph theory

In the traditional setting of spatial games, the individuals of a population are arranged on a regular lattice, and interactions occur among nearest neighbours (Nowak & May 1992). In evolutionary graph theory, the individuals occupy the vertices of a graph, and the edges denote who interacts with whom (Lieberman *et al.* 2005; Ohtsuki & Nowak 2006*a*,*b*; Ohtsuki *et al.* 2006; Pacheco *et al.* 2006; Szabó & Fath 2007; Lehmann *et al.* 2007; Taylor *et al.* 2007*a*,*b*; Fu *et al.* 2009; Santos *et al.* 2008). Spatial games are a special case of evolutionary graph theory. Also, the well-mixed population simply corresponds to the special case of a complete graph with identical weights. Note that the interaction graph and the replacement graph need not be identical (Ohtsuki *et al.* 2007), but we do not discuss this extension in the present paper.

Evolutionary dynamics on graphs depend on the update rule. Many different update rules can be considered, but here we limit ourselves to ‘death–birth’ (DB) updating: one individual is chosen at random to die; the neighbours compete for the empty site proportional to fitness. This update rule can also be interpreted in terms of social learning: a random individual decides to update his strategy; then he chooses among his neighbours' strategies proportional to fitness. All results of this section (unless otherwise stated) hold for the limit of weak selection and low mutation.

### (a) Structural dominance for two strategies

**Figure 1.**
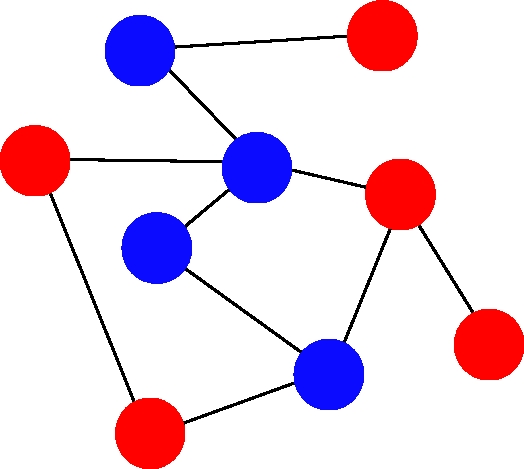
In evolutionary graph theory, the individuals of a population occupy the vertices of a graph. The edges denote who interacts with whom—both for accumulating payoff and for reproductive competition. Here, we consider two strategies, *A* (blue) and *B* (red). Evolutionary dynamics on graphs depend on the update rule. In this paper, we use death–birth updating: a random individual dies; the neighbours compete for the empty site proportional to fitness.

At first, we consider games between two strategies, *A* and *B*, given by the payoff matrix (2.1). Each individual interacts with all of its neighbours and thereby accumulates a payoff (figure 1). Individual *i* has payoff *F*_*i*_ and fitness *f*_*i*_ = 1 + *wF*_*i*_, where again *w* is the intensity of selection. The limit of weak selection is given by *w* → 0.

For regular graphs, we can calculate the *σ* parameter. A graph is regular if all individuals have the same number, *k*, of connections. This number is called the degree of the graph. The family of regular graphs includes many spatial lattices and also random regular graphs. For large population size, *N* ≫ *k*, Ohtsuki *et al.* (2006) found4.1
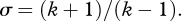



For general heterogeneous graphs such as Erdos–Renyi random graphs or scale-free networks, we do not have analytical results. Computer simulations suggest that in some cases, the results of regular graphs carry over, but *k* is replaced by the average degree *k̄*. Thus, there is some indication that *σ* = (*k̄* + 1)/(*k̄* − 1). This result seems to hold as long as the variance of the degree distribution is not too large (Ohtsuki *et al.* 2006).

For one particular heterogeneous graph, we have an exact result. The star is a structure where one individual is in the hub and *N* − 1 individuals populate the periphery. The average degree is *k̄* = 2(*N* − 1)/*N*, but the variance is large; hence, we do not expect equation (4.1) to hold. Tarnita *et al* (2009*a*) showed that *σ* = 1 for DB updating on a star for all population sizes, *N* ≥ 3, and any mutation rate.

### (b) The replicator equation on graphs and evolutionary stability

The deterministic dynamics of the average frequencies of strategies on regular graphs can be described by a differential equation (Ohtsuki & Nowak 2006*b*). This equation has the structure of a replicator equation, but the graph induces a transformation of the payoff matrix. The replicator equation on graphs is of the form4.2
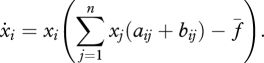

Here, *x*_*i*_ denotes the relative abundance (= frequency) of strategy *i*. There are *n* strategies. The payoffs are given by the *n* × *n* matrix *A* = [*a*_*ij*_]. The parameter *f̄* denotes the average fitness of the population as in the standard replicator equation. The *B* = [*b*_*ij*_] matrix is anti-symmetric and captures the essence of local competition on a graph, where it matters how much strategy *i* gets from *i* and *j* and how little *j* gets from *i* and *j*. For DB updating, we have4.3
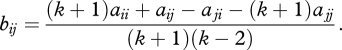



An immediate consequence of the replicator equation on graphs is a concept of ESS in graph structured populations (Ohtsuki & Nowak 2008). A strategy is evolutionarily stable if it can resist invasion by infinitesimally small fractions of other strategies (Maynard Smith 1982). Let us use equations (4.2) and (4.3) for a game between two strategies *A* and *B* given by the payoff matrix (2.1). We obtain the following ESS condition:4.4
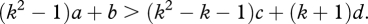



This condition has a beautiful geometric interpretation. For evolutionary stability, we ask if a homogeneous population of *A* individuals can resist the invasion by a small fraction of *B* individuals. Because of weak selection, the fitness of the invaders is roughly the same as that of the residents. Therefore, in the beginning about half of all invaders die out while the other half reproduce. Weak selection leads to a separation of two time scales: (i) on a fast time scale, there is a local equilibration, leading to an ‘invasion cluster’; (ii) on a slow time scale, the frequency of the invaders changes (either up or down). The invasion cluster has geometric properties which determine the ESS conditions. The essential property is the following: a random ensemble of neighbours around one *B* individual contains on average one *B* individual. Hence, the invasion cluster forms a half line of *B* individuals. The ESS condition specifies that the tip of the half line shrinks.

### (c) Structural dominance for *n* strategies on graphs

The replicator equation on graphs suggests an extension of the concept of structural dominance (of §3) to games with *n* strategies for low mutation. If we use the modified payoff matrix, *A* + *B*, for equation (2.4) we obtain4.5
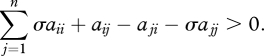



Here *σ* = (*k* + 1)/(*k* − 1) as it should be. We will show in a forthcoming paper that such a condition holds for games with *n* strategies for a wide variety of population structures and update rules (for low mutation and weak selection).

## 5. Games in phenotype space

Typically, individuals express other phenotypic properties in addition to their behavioral strategies. These phenotypic properties can include size, height, other aspects of physical appearance or other behaviours. Let us consider a situation where the behavioural strategies are conditional on these other phenotypic properties. A particular setting was studied by Antal *et al.* (2009*c*): there are two strategies, *A* and *B*, and the standard payoff matrix (2.1); the phenotype is given by one (or several) continuous or discrete variables. Individuals only interact with others who have the same phenotype. Reproduction is proportional to fitness. Offspring inherit the strategy and the phenotype of their parent subject to mutation. The population drifts in phenotype space. Occasionally, the population splits into two or several clusters, but in the long run the population remains localized in phenotype space, because of sampling effects that occur in finite populations.

**Figure 2.**
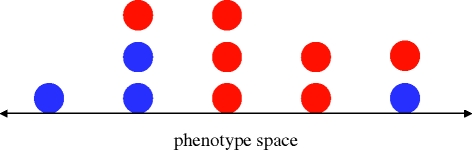
We study games in a one-dimensional, discrete phenotype space. The phenotype of an individual is given by an integer *i*. The offspring of this individual has phenotype *i* − 1, *i*, *i* + 1 with probabilities *v*, 1 − 2*v*, *v*, where *v* is the phenotypic mutation rate. Offspring also inherit the strategy of their parent (red or blue) with a certain mutation rate. Each individual interacts with others who have the same phenotype and thereby derives a payoff. The population drifts through phenotype space. Strategies tend to cluster. For evolution of cooperation, this model represents a very simple scenario of tag-based cooperation (or ‘Green beard’ effects).

Antal *et al.* (2009*c*) developed a general theory that is based on calculating the coalescent probabilities among individuals. They also perform specific calculations for a one-dimensional phenotype space (figure 2). The phenotypic mutation rate is *v*. If the phenotype of the parent is given by the integer *i*, then the phenotype of the offspring is given by *i* − 1, *i*, *i* + 1 with probabilities *v*, 1 − 2*v*, *v*, respectively. For weak selection, *A* is more abundant than *B* if a *σ*-type condition (3.1) holds. For large population size and low strategy mutation, the structural coefficient is given by5.1
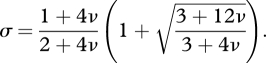



Here, *ν* = 2*Nv*, where *N* is the population size. Note that *σ* is an increasing function of *ν*. For large *ν*, we have 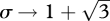.

When applied to the evolution of cooperation, games in phenotype space are related to models for tag-based cooperation (Riolo *et al.* 2001; Traulsen & Claussen 2004; Jansen & van Baalen 2006; Traulsen & Nowak 2007) or ‘Green beard effects’. The model of Antal *et al.* (2009*c*) is the simplest model of tag-based cooperation that leads to the evolution of cooperation without any additional assumptions such as physical spatial structure.

## 6. Evolutionary set theory

The geometry of human populations is determined by the associations that individuals have with various groups or sets. We participate in activities or belong to institutions where we interact with other people. Each person belongs to several sets. Such sets can be defined, for example, by working for a particular company or living in a specific location. There can be sets within sets. For example, the students of the same university study different subjects and take different classes. These set memberships determine the structure of human society: they specify who meets whom, and they define the frequency and context of meetings between individuals.

**Figure 3.**
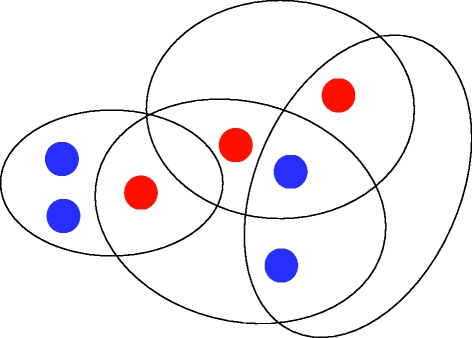
In evolutionary set theory, the individuals of a population are distributed over sets. Individuals interact with others who are in the same set. If two individuals share several sets, they interact several times. The interactions lead to payoff in terms of an evolutionary game. Strategies and set memberships of successful individuals are imitated. There is a strategy mutation rate and a set mutation rate. The population structure becomes effectively well mixed if the set mutation rate is too low or too high. There is an intermediate set mutation rate which maximizes the clustering of individuals according to strategies. Evolutionary set theory is a dynamical graph theory. The population structure changes as a consequence of evolutionary updating.

Tarnita *et al.* (2009*b*) proposed a framework of population structure called ‘evolutionary set theory’ (figure 3). A population of *N* individuals is distributed over *M* sets. Each individual belongs to exactly *K* sets. Interactions occur within a given set. If two people have several sets in common, they interact several times. Interaction between individuals leads to a payoff from an evolutionary game. Let us consider a game between two strategies *A* and *B* given by payoff matrix (2.1).

Both the strategy and the set memberships are subject to evolutionary updating. Successful individuals are imitated with respect to their behaviour and their set associations. Hence, successful strategies spread and successful sets attract more members. There is a strategy mutation rate and a set mutation rate. The set mutation leads people to explore new sets independent of imitation events. There is migration between sets because of both imitation of other people and set mutation. The stochastic mutation–selection process generates a stationary distribution of the population over sets and strategies.

For weak selection, *A* is more abundant than *B* if a *σ*-type inequality (3.1) holds. Tarnita *et al.* (2009*b*) calculated the exact *σ*, which depends on the population size, *N*, the number of sets, *M*, the number of set memberships, *K*, the set mutation rate, *v*, and the strategy mutation rate, *u*. A simple expression is obtained if we assume large population size and low strategy mutation rate6.1
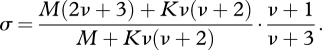



Here, we use *ν* = 2*Nv*.

The parameter *σ* is an increasing function of *M* and a one-humped function of *v*. If *v* is too small, then the entire population clumps in the same sets. If *v* is too large, then the membership of individual sets does not persist long enough in time. In both cases, the population is essentially ‘well mixed’ (which means *σ* → 1). There is an intermediate optimum value of *ν* which is approximately given by 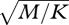. For this value of *ν*, we obtain the maximum value of *σ*, which is also close to 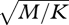. Larger values of *σ* are obtained if there are many sets, *M*, and each person can only be in very few of them. The minimum number of set memberships is *K* = 1.

In an extension of the model, individuals interact with others only if they have at least *L* sets in common (Tarnita *et al.* 2009*b*). More specifically, two individuals interact *i* times if they have *i* ≥ *L* sets in common; otherwise, they do not interact at all. In this case, the same equations apply as before but *K* is replaced by K* which is given by6.2
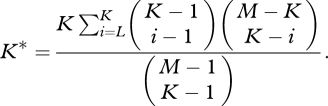



Note that K* can be less than one. Now, it is no longer the case that the maximum *σ* is obtained for *K* = 1. Instead, for a given *M* the maximum *σ* is obtained for *L* = *K* = *M*/2, which maximizes the combinatorial possibilities of social identities.

## 7. Evolution of cooperation by ‘spatial selection’

We can now use the results of the previous sections to study how population structure affects the evolution of cooperation. The most difficult setting for the evolution of cooperation is given by the prisoner's dilemma. The aspects of cooperation can also be found in other games, but they represent somewhat relaxed situations. In this section, we focus on the prisoner's dilemma.

### (a) The prisoner's dilemma

The prisoner's dilemma is a game with two strategies, cooperation, *C* and defection, *D*. If two cooperators meet they get payoff, *R*. If two defectors meet, they get a lower payoff, *P*. But if a cooperator meets a defector, the defector gets the highest payoff, *T*, while the cooperator gets the lowest payoff, *S*. We have *T* > *R* > *P* > *S*. The payoff matrix is given by7.1
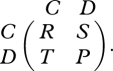



In a well-mixed population, cooperators are less abundant than defectors in the stationary distribution of the mutation–selection process, because *R* + *S* < *T* + *P*. But in a structured population with *σ* > 1, this situation can be reversed: if *σ* > (*T* − *S*)/(*R* − *P*) > 1, then cooperators are more abundant than defectors in the stationary distribution for weak selection. The *σ* values of §§4–6 provide the exact conditions for the evolution of cooperation in the respective models. The larger the value of *σ* the broader is the parameter range that is still compatible with the evolution of cooperation. For DB updating on regular graphs, the largest *σ* value is given for the cycle where *σ* = 3. This means that DB updating on regular graphs can support cooperation as long as 3 > (*T* − *S*)/(*R* − *P*) > 1. For games in a one-dimensional phenotype space, cooperation is possible for 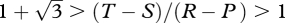. For evolutionary set theory, using the optimum set mutation rate, we find 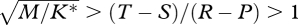. Here, *M* is the number of sets and K* is an effective number of set memberships as given by equation (6.2). Therefore, evolutionary set theory can lead to unbounded values of *σ*.

Using the replicator equation on graphs (4.2) and (4.3) for payoff matrix (7.1) shows that all four dynamical scenarios are possible depending on parameter choices: (i) cooperators dominate defectors; (ii) cooperators and defectors coexist; (iii) cooperators and defectors are bistable; or (iv) defectors dominate cooperators (Taylor & Nowak 2007). In particular, cooperators are evolutionarily stable against invasion by defectors if (*T* − *R* − *P* + *S*) + (*T* − *P*)*k* − (*T* − *R*)*k*^2^ > 0.

### (b) Costs and benefits—the simplified game

A simplified prisoner's dilemma is obtained if cooperators pay a cost, *c*, for others to receive a benefit, *b*, while defectors pay no costs and distribute no benefits. The payoff matrix is given by7.2
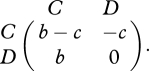



The game is a prisoner's dilemma if *b* > *c* > 0. Using the *σ* factors of §§4–6, we obtain the following conditions for the evolution of cooperation under weak selection:

— For DB updating on graphs, we have (Ohtsuki *et al.* 2006)7.3
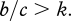

— For games in a one-dimensional phenotype space, we have (Antal *et al.* 2009*c*)7.4
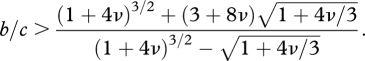

The critical benefit-to-cost ratio is a declining function of the phenotypic mutation rate, *ν*. For large values of *ν*, it converges to the simple expression 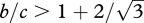.
— For evolutionary set theory, we have (Tarnita *et al.* 2009*b*)7.5
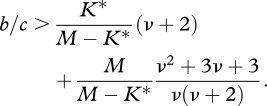

The critical benefit-to-cost ratio is a declining function of the ratio *M*/K*. Moreover, it is a U-shaped function of the set mutation rate, *ν*. For the optimum value of the set mutation rate (and *M* ≫ K*), we obtain 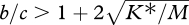.

The relationship between the critical benefit-to-cost ratio, (*b*/*c*)*, and the structure coefficient, *σ*, is given by (*b*/*c*)* = (*σ* + 1)/(*σ* − 1). For calculating *σ*, it is enough to know (*b*/*c*)* or vice versa (Tarnita *et al.* 2009*a*). Hence, if we only want to know which of the two strategies is more abundant in the stationary distribution for weak selection, it is enough to consider the simplified payoff matrix (7.5) and from there we can calculate *σ*. But for a general analysis of evolutionary game dynamics, it is of course not enough to study the simplified matrix. For example, using the replicator equation on graphs, we find that cooperators dominate defectors for *b/c* > *k*, while defectors dominate cooperators for *b/c* < *k*. Hence, only two of the four dynamical scenarios can occur, while all four dynamical scenarios are possible for the general prisoner's dilemma (§7*a*).

### (c) Spatial selection is distinct from group selection and kin selection

The mechanism for the evolution of cooperation (Nowak 2006*a*,*b*) that is operating in all models that we have discussed here could be called spatial selection. Cooperators prevail because they can form clusters, either in physical space, on networks, in phenotype space or in sets. Individuals within such clusters gain a higher payoff than defectors that try to invade them. Spatial selection can favour cooperation if the structure coefficient, *σ*, exceeds one.

Spatial selection is a different mechanism than group selection (Wynne-Edwards 1962; Wilson 1975, 1983; Wade 1977, 1978; Uyenoyama 1979; Leigh 1983; Szathmáry & Demeter 1987; Goodnight 1990*a*,*b*; Goodnight & Stevens 1997; Boyd & Richerson 2002; Kerr & Godfrey-Smith 2002; Paulsson 2002; Fletcher & Zwick 2004; Wilson & Hölldobler 2005; Killingback *et al.* 2006; Traulsen & Nowak 2006; Reeve & Hölldobler 2007; Traulsen *et al.* 2008). For group selection, we have competition on two different levels: individuals compete within groups and groups compete with each other. For spatial selection, there is only clustering of individuals and no second level of selection. Consequently, the underlying mathematical theories are different, although the structural dominance condition (3.1) applies in both cases (Tarnita *et al.* 2009*a*).

Kin selection can arise if evolutionary games occur between genetical relatives. It is a mechanism for the evolution of cooperation if there is conditional behaviour based on kin recognition. For example, Haldane would jump into the river to save two brothers or eight cousins. In such a setting, it is clear that kin selection is different from group selection and different from spatial selection. Furthermore, the latter mechanisms can also operate in the context of cultural evolution where successful strategies spread by imitation and learning in the absence of any genetic reproduction.

The mathematical methods of inclusive fitness theory have led to interesting results over the years (Taylor 1992*a*,*b*; Taylor *et al.* 2000, 2007*a*,*b*; Rousset 2004) and provide a useful complement to other approaches. But some authors claim that kin selection is a universal mechanism for the evolution of cooperation (Lehmann & Keller 2006; West *et al.* 2007). Central to this claim is the idea that Hamilton's rule is always true. Hamilton's rule states that cooperation is favoured over defection if *b/c* > 1/*r*, where *r* is ‘relatedness’. We think that this universality claim of kin selection theory is wrong for several reasons. The general question of evolution of cooperation cannot be studied by a model that only works with costs and benefits. The simplified payoff matrix (7.2) represents only a special case of matrix (7.1). Furthermore, inclusive fitness calculations are always formulated for weak selection and vanishing mutation. They analyze which of two arbitrarily close strategies is marginally more abundant on average. There is no attempt to characterize any evolutionary dynamics or to study the interaction of more than two strategies. Finally, the over-generalization of kin selection theory causes the relatedness parameter, *r*, in Hamilton's rule to become an undefined quantity, which can be freely adjusted to fit every new situation. Already for simple models, *r* differs from any empirical concept of pedigree relatedness.

The claim that relatedness is the fundamental reason for all evolution of cooperation is a mistake of cause and effect. All mechanisms for the evolution of cooperation can be seen as leading to assortment of cooperation and defection, but assortment itself is not a mechanism. It is the consequence of a mechanism. The key question is always how assortment is achieved. The dogmatic insistence on relatedness obscures a useful discussion of ‘mechanism’.

For other criticism of the universality claim of kin selection, see Wilson (2005, 2008), Wilson & Hölldobler (2005), Fletcher *et al.* (2006), Wild & Traulsen (2007), Fletcher & Doebeli (2009) and Van Veelen (2009).

## 8. Discussion

At the centre of every evolutionary process is a population of reproducing individuals. The structure of that population affects evolutionary dynamics. In a well-mixed population, natural selection could favour one strategy, but in a structured population, another strategy might win. Changing the underlying population structure can reverse the outcome of an evolutionary process (Nowak & May 1992).

We began by showing some results for stochastic evolutionary game dynamics in well-mixed populations of finite size. Inequalities (2.3)–(2.6) specify which strategies are more abundant in the equilibrium distribution of the mutation–selection process. They can be used for any *n × n* payoff matrix, and they provide an immediate answer to the question of which strategy is favoured in the limit of weak selection. These conditions can be more informative than the traditional Nash or ESS conditions if we want to understand evolutionary dynamics in finite populations.

Next, we introduced the concept of structural dominance (Tarnita *et al.* 2009*a*). For almost any evolutionary process in a structured population, strategy *A* is favoured over *B* for weak selection if *σa* + *b* > *c* + *σd*. Here, *a*, *b*, *c*, *d* are the entries of the payoff matrix (2.1) and *σ* is the structure coefficient. For a large well-mixed population, we have *σ* = 1, which reduces structural dominance to the well-known concept of risk dominance, *a* + *b* > *c* + *d*. But for many population structures and update rules, the structure coefficient can deviate from one. If *σ* > 1, then the diagonal entries of the payoff matrix are emphasized over the off-diagonal entries. This means that the population structure leads to a clustering of strategies, where individuals who have the same strategy are more likely to interact (Nowak & May 1992). This positive assortment of strategies, however, does not always occur; for some population structures and update rules, we obtain *σ* = 1 as if the population was well mixed. For example, birth–death updating on any regular graph leads to *σ* = 1 (Ohtsuki *et al.* 2006), while DB updating on a star leads to *σ* = 1 for any mutation rate.

There is (as yet) no general mathematical framework that would encompass evolutionary dynamics for any kind of population structure. We have discussed three different approaches: evolutionary graph theory, games in phenotype space and evolutionary set theory.

In evolutionary graph theory, the individuals of a population occupy the vertices of a graph, and the edges determine who interacts with whom. Evolutionary graph theory is a generalization of the earlier models of spatial games to larger classes of population structure. This extension seems to be useful for studying human populations where the social network determines the patterns of interaction. The graph is usually fixed on the time scale of evolutionary updating, which is an important limitation of the existing theory, although some models with dynamical graphs have been proposed (Pacheco *et al.* 2006). For evolutionary dynamics on fixed, regular graphs, we can calculate the structure coefficient for various update rules. We can derive a replicator equation on graphs (Ohtsuki & Nowak 2006*a*,*b*), discuss evolutionary stability (Ohtsuki & Nowak 2008) and calculate fixation probabilities (Ohtsuki *et al.* 2006). There can also be different graphs for interaction and replacement (Ohtsuki *et al.* 2007).

For games in phenotype space, we have explored the idea that the strategic behaviour is dependent on other phenotypic properties. We observe the clustering of strategies in phenotype space. The population structure affects the strategic behaviour, but evolutionary updating occurs as for a well-mixed population. We call this approach ‘global updating’. This is the key difference between games in phenotype space and evolutionary graph theory as presented here.

The structure of human society can be described by set memberships. We belong to many sets and are more likely to interact with those who are in the same sets. In evolutionary set theory, the individuals of the population are distributed over sets. Each individual can belong to several sets. Individuals interact with others in the same set. Two people can have more than one set in common. The evolutionary updating includes both strategy and set memberships. Successful strategies breed imitators, successful sets attract more members. Evolutionary set theory offers a particular approach for a dynamical graph theory. At any one time, the structure of the population can be described by a graph, but this graph changes under evolutionary updating.

Many of our results hold only for weak selection: the payoff that is earned in the game makes a small contribution to the total fitness of an individual. We think that such an assumption is useful for human interactions. It is rarely the case that all our stakes are in one game. Therefore, our social instincts might well be adapted to the situations of weak selection; but it is an important goal to derive simple results that hold for any intensity of selection. Some success in this direction has already been achieved (Traulsen *et al.* 2008; Antal *et al.* 2009*a*).

We hope that the structural frameworks presented here will turn out to be useful for studying the social evolutionary dynamics of humans. Every day, we learn from each other and adjust our strategies. We are embedded in social structures that determine the frequency and context of interactions. We compete with others and have to find ways to cooperate. Our results have implications for the evolution of cooperation. Evolutionary dynamics on graphs, in sets and in phenotype space can favour cooperators, because they cluster in physical or other spaces.

When discussing human behaviour, let us keep in mind that we are never outside of the frameworks of direct or indirect reciprocity. Our actions tend to be conditional on previous experience. Direct reciprocity occurs when my behaviour towards you depends on what you have done to me. Indirect reciprocity means my behaviour towards you also depends on what you have done to others. Eventually, direct and indirect reciprocity must be combined with the frameworks that are presented here in order to obtain a complete mathematical theory of social evolutionary dynamics of humans.
